# Identifying frailty in trials: an analysis of individual participant data from trials of novel pharmacological interventions

**DOI:** 10.1186/s12916-020-01752-1

**Published:** 2020-10-22

**Authors:** Peter Hanlon, Elaine Butterly, Jim Lewsey, Stefan Siebert, Frances S. Mair, David A. McAllister

**Affiliations:** 1 Institute of Health and Wellbeing, University of Glasgow, 1 Lilybank Gardens, Glasgow, G12 8RZ UK; 2 Institute of Infection, Immunity and Inflammation, University of Glasgow, Glasgow, UK

**Keywords:** Frailty, Randomised controlled trials, Diabetes mellitus, Rheumatoid arthritis, Chronic obstructive pulmonary disease

## Abstract

**Background:**

Frailty is common in clinical practice, but trials rarely report on participant frailty. Consequently, clinicians and guideline-developers assume frailty is largely absent from trials and have questioned the relevance of trial findings to frail people. Therefore, we examined frailty in phase 3/4 industry-sponsored clinical trials of pharmacological interventions for three exemplar conditions: type 2 diabetes mellitus (T2DM), rheumatoid arthritis (RA), and chronic obstructive pulmonary disease (COPD).

**Methods:**

We constructed a 40-item frailty index (FI) in 19 clinical trials (7 T2DM, 8 RA, 4 COPD, mean age 42–65 years) using individual-level participant data. Participants with a FI > 0.24 were considered ‘frail’. Baseline disease severity was assessed using HbA1c for T2DM, Disease Activity Score-28 (DAS28) for RA, and % predicted FEV1 for COPD. Using generalised gamma regression, we modelled FI on age, sex, and disease severity. In negative binomial regression, we modelled serious adverse event rates on FI and combined results for each index condition in a random-effects meta-analysis.

**Results:**

All trials included frail participants: prevalence 7–21% in T2DM trials, 33–73% in RA trials, and 15–22% in COPD trials. The 99th centile of the FI ranged between 0.35 and 0.45. Female sex was associated with higher FI in all trials. Increased disease severity was associated with higher FI in RA and COPD, but not T2DM. Frailty was associated with age in T2DM and RA trials, but not in COPD. Across all trials, and after adjusting for age, sex, and disease severity, higher FI predicted increased risk of serious adverse events; the pooled incidence rate ratios (per 0.1-point increase in FI scale) were 1.46 (95% CI 1.21–1.75), 1.45 (1.13–1.87), and 1.99 (1.43–2.76) for T2DM, RA, and COPD, respectively.

**Conclusion:**

The upper limit of frailty in trials is lower than has been described in the general population. However, mild to moderate frailty was common, suggesting trial data may be harnessed to inform disease management in people living with frailty. Participants with higher FI experienced more serious adverse events, suggesting screening for frailty in trial participants would enable identification of those that merit closer monitoring. Frailty is identifiable and prevalent among middle-aged and older participants in phase 3/4 drug trials and has clinically important safety implications.

## Background

As average life expectancy rises and multimorbidity increases [1], frailty is becoming an increasingly important consideration in the management of chronic disease [2]. Frailty describes a clinical state of decreased function across multiple physiological systems characterised by vulnerability to adverse health outcomes and decompensation in response to physiological stress [2]. A large number of measures exist to identify and quantify frailty; however, two models have dominated the literature: the frailty index and the frailty phenotype [2, 3]. The frailty index (FI) is based on a ‘cumulative deficit’ model wherein deficits including long-term conditions, symptoms, functional impairments, and laboratory abnormalities are counted [4]. Larger deficit counts indicate a greater degree of frailty. The main alternative to the FI, the frailty phenotype, identifies frailty where three of the following five specific criteria are met, unintentional weight loss, weakness, slow gait speed, exhaustion, and low physical activity [5]. Although distinct concepts, there is considerable overlap in the populations identified by the frailty index and frailty phenotype [6]. Both approaches have been widely validated and associated with adverse health outcomes including mortality, hospitalisation and disability [2].

Managing chronic illness in people living with frailty is challenging [2, 7], not least because randomised controlled trials, which (via clinical guidelines) underpin safe and effective management, are said to exclude people with frailty [7–9]. As such, the applicability of trial findings to people living with frailty is not clear. This leaves clinicians uncertain about treatment effectiveness, which further complicates management of patients whose care is already complex and challenging.

Despite these concerns, direct evidence concerning frailty in clinical trials is scarce. Very few trials have measured frailty. Considering drug trials specifically, we found three (the HYVET and SPRINT studies of hypertension [10, 11] and TOPCAT study of heart failure [12]) which performed post hoc analysis of frailty using the frailty index and a fourth (TRILOGY ACS for unstable angina), which assessed frailty using the frailty phenotype model in a subset of participants aged over 65 years [13]. Frailty was found to be prevalent in these trials, but as all four specifically targeted older people, it is not known whether frailty may also be found in the much larger and more influential body of trials not specifically targeted at older people. More recently, a pooled analysis of 14 cardiovascular trials in older people (153,696 participants, mean age 70.8 years) showed that a frailty index was associated with all-cause and cardiovascular mortality, as well as cardiovascular events [14].

Hitherto, inferences about trial representativeness have largely been based on the observation that, on applying trial eligibility criteria to routine electronic health records, ineligible patients are older and frailer and have more comorbidities [15]. Recently, however, on directly measuring comorbidities using individual-level participant data (IPD) in 116 industry-funded trials, we found that multimorbidity was common in trial participants [16]. Although frailty is associated with multimorbidity, it is a distinct entity [1] and it is not clear whether frailty is also common among trial participants. Moreover, since trial IPD contains rich data on physiological status (e.g. albumin, haemoglobin, body mass index), symptoms (e.g. breathlessness, fatigue), and function (e.g. impaired mobility), there is the potential to measure frailty.

In this study, we use IPD from existing clinical trials for three exemplar chronic conditions (type 2 diabetes mellitus (T2DM), rheumatoid arthritis (RA), and chronic obstructive pulmonary disease (COPD)) to construct a frailty index. We then quantify the prevalence of frailty in these clinical trial populations and examine whether frailty is associated with serious adverse events in the clinical trials studied.

## Methods

### Study design and participants

IPD from industry-sponsored clinical trials were identified from two repositories: Clinical Study Data Request (CSDR) and the Yale University Open Data Access (YODA) project. Trials were selected according to a pre-specified protocol (Prospero CRD42018048202) as part of a wider project assessing multimorbidity in clinical trials. Trials were eligible for inclusion if they evaluated pharmacological treatments for a long-term condition; were registered with clinicaltrials.gov; started after 1 January 1990; were phase 2/3, 3, or 4; included ≥ 300 participants; and had an upper age limit ≥ 60 years (or no maximum). From this wider set of trials, we selected three exemplar conditions (T2DM, RA, and COPD) in which to assess frailty. These conditions were chosen as frailty is common, and has been shown to affect younger people, in the context of these chronic conditions [1, 17–19]. Furthermore, frailty is a clinically relevant concept in the management of these conditions, having been highlighted as an important factor influencing treatment targets [20, 21].

### Procedures

We measured frailty using a frailty index approach, based on the cumulative deficit model of frailty described by Rockwood and Mitnitski [22]. A frailty index is a count of health-related deficits (including long-term health conditions, laboratory abnormalities, symptoms, and functional limitations) across a range of physiological and mental health domains. Each individual’s frailty index value is calculated as the sum of deficits present, divided by the total number of deficits in the frailty index. For example, an individual with 8 out of a possible 40 deficits would have a frailty index of 0.2 (8/40). The frailty index was used to measure frailty in the HYVET, SPRINT and TOPCAT trials, is applicable at any age [10–12, 23–25], and can be calculated from any dataset where a sufficient number of deficits is recorded. It is therefore suitable for measuring frailty in our set of trials.

A standard procedure exists for selecting variables for inclusion as ‘deficits’ in a frailty index [26]. Deficits should (i) be associated with age and poorer health, (ii) cover a range of physiological areas (e.g. physiological measures, physical function, long-term conditions from different organ systems), and (iii) be neither ubiquitous in the target population nor be very rare (e.g. < 1% prevalence in the target population). We applied these criteria to possible deficits identified from trial baseline data. Existing literature was used to judge if a deficit met the above criteria.

Symptoms and functional measures were identified using baseline quality of life and symptom questionnaires. We used the same deficits for all trials within each index condition. Deficits differed between index conditions as different questionnaires were used in the respective trials. Laboratory and anthropometric deficits (e.g. blood pressure, body mass index) were identified from baseline values. We excluded from the frailty index any deficit with > 5% missing data. To assess if any variables were strongly correlated, we analysed all pairs of deficits using the Pearson and Spearman’s rank correlation coefficients (for pairs of binary and categorical deficits, respectively). Where there was high correlation (> 0.3), only one of the correlated variables was included in the frailty index [27]. For each index condition, we identified 40 deficits to be included in the frailty index (supplementary appendix). Participants with complete data for at least 38 of these deficits were included in the analysis. The frailty index was calculated as the total number of deficits present divided by the total number of deficits with complete data for that individual.

We had intended to use medical history data to identify long-term conditions, but this was frequently redacted (as a privacy measure) or not recorded. We therefore identified long-term conditions based on concomitant medications, using definitions we have previously published [16].

### Outcomes

Applying cut-off values to define frailty has proved controversial, with no consensus on a value above which a person should be identified as living with frailty. We therefore report the entire distribution of the frailty index for each trial. We also separately described the distribution in trial participants above 65 years. To facilitate comparison with the published literature, we also categorised the frailty index into no frailty (< 0.12), mild (0.12–0.24), moderate (0.24–0.36), and severe frailty (> 0.36), based on cut-points used in the electronic frailty index (used in routine clinical practice) [27].

We assessed the relationship between frailty index and the following baseline characteristics: age, sex, severity of the index condition, and long-term condition count. We assessed severity of T2DM by measuring glycated haemoglobin (HbA1c) as a proxy marker, while in RA we used the Disease Activity Score in 28 joints (DAS28) and for COPD the forced expiratory volume in 1 s as a percentage of predicted value (% predicted FEV1).

Finally, we assessed whether the frailty index at baseline predicts serious adverse events during trial follow-up. Trials record all adverse events occurring during the trial period regardless of their relationship (or lack of relationship) with the trial treatment. Certain adverse events are characterised as ‘serious adverse events’ (SAEs). SAEs are those meeting one or more of the following criteria: (i) results in death, (ii) is life threatening, (iii) results in hospitalisation, (iv) results in persistent or significant disability/incapacity, or (v) is a congenital abnormality/birth defect.

### Statistical analysis

All analyses were conducted according to a pre-specified protocol.

All trial data were held within secure repositories that only permit export of aggregate, non-identifiable data. Therefore, to allow full description of the distribution of the frailty index for each trial while avoiding the risk of disclosure, we used statistical distributions to represent the frailty index. For each trial, we fitted the frailty index to each of the following distributions: lognormal, gamma, Weibull, and generalised gamma. We then compared the fit of each distribution using Kolmogorov-Smirnov tests (*p* > 0.05 taken as good fit, failing to reject the null hypothesis that the distributions were different). The generalised gamma distribution was found to fit the frailty index distribution well for all trials. Parameters describing the distribution for each trial were exported from the secure environments to allow us to report the distribution of the frailty index for each trial. We calculated the frailty distribution for the whole trial population. We then repeated the process restricting the trial population to people over 65 years.

We then modelled frailty index on age, sex, and disease severity using generalised gamma regression models. Each trial was modelled separately. Non-linear relationships between age, disease severity, and frailty index were explored using fractional polynomials. There was no improvement in model fit incorporating non-linear terms. The coefficients and variance-covariance matrices from these models were exported from the secure environments to allow us to report the mean frailty index for specific age, sex, and disease severity combinations.

Within the secure environments, we fitted negative binomial models of serious adverse event rates on frailty index, age, sex, and disease severity, exporting the coefficients and variance covariance matrices as before. For each index condition, we performed random-effects meta-analysis (using inverse variance weighting) to obtain overall estimates of the associations between serious adverse events and frailty index (adjusted for age, sex, and disease severity).

Data processing and analysis was performed using R (version 3.6.1). Meta-analyses were performed using RevMan5. All model outputs are available in the supplementary appendix.

## Results

### Identification of studies

**Fig. 1:**
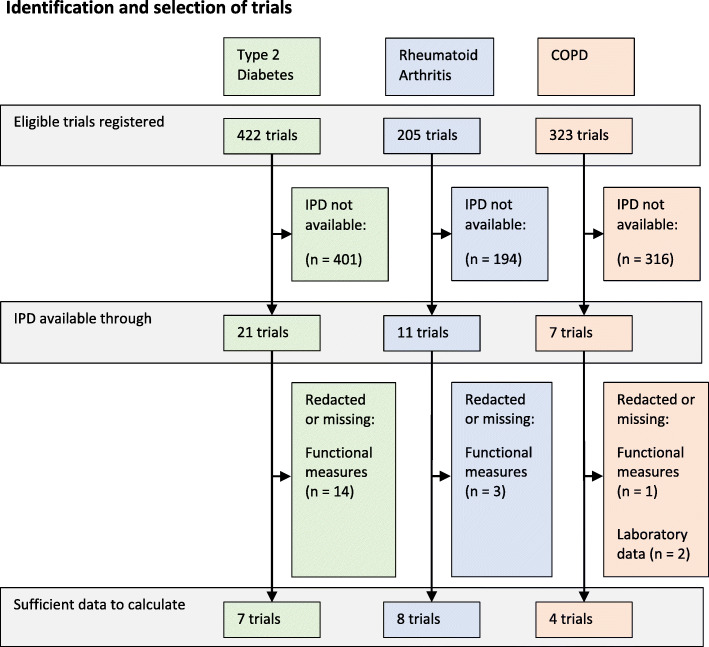
Flow diagram of identification of trial individual participant data and inclusion in analysis

We identified 39 trials meeting our inclusion criteria for which IPD were available in the CSDR or YODA repositories. Of these, 19 trials (7 T2DM trials, 8 RA trials, and 4 COPD trials) contained IPD on a range of variables sufficient to calculate the frailty index. In the remaining 20 trials, data on functional deficits and/or laboratory measures were either redacted or not reported. The selection of trials is summarised in Fig. 1. The characteristics of the included trials are summarised in Table 1.

**Table 1:** Details of included studies

Trial ID	Sponsor	Drug	Comparison	Phase	Total participants	Age eligibility	*N* (%) aged ≥ 65	Mean (SD) age	N (%) female
T2DM									
NCT01106625	Janssen	Canagliflozin	Placebo	3	469	18–80 years	84 (17%)	56.7 (9.3)	230 (40%)
NCT01106677	Janssen	Canagliflozin	Placebo	3	1284	18–80 years	206 (16%)	55.4 (9.4)	679 (53%)
NCT00734474	Lilly	Dulaglutide	Sitagliptin, Placebo/Sitagliptin	3	1202	18–75 years	364 (30%)	54.1 (9.9)	643 (54%)
NCT01064687	Lilly	Dulaglutide	Exenatide, Placebo	3	978	≥ 18 years	197 (20%)	55.7 (9.8)	406 (42%)
NCT01075282	Lilly	Dulaglutide	Insulin	3	810	≥ 18 years	188 (23%)	56.7 (9.5)	393 (49%)
NCT01191268	Lilly	Dulaglutide	Insulin	3	884	≥ 18 years	280 (32%)	59.3 (9.2)	411 (57%)
NCT01624259	Lilly	Dulaglutide	Liraglutide	3	599	≥ 18 years	138 (23%)	56.7 (9.3)	312 (52%)
RA									
NCT00236028	Janssen	Infliximab	Methotrexate	3	1036	18–75 years	127 (12%)	50 (12.6)	733
NCT00264537	Janssen	Golimumab	Placebo	3	637	≥ 18 years	52 (8%)	49.5 (12.2)	528 (83%)
NCT00264550	Janssen	Golimumab	Placebo	3	444	≥ 18 years	38 (9%)	50.4 (11.3)	358 (81%)
NCT00361335	Janssen	Golimumab	Placebo	3	643	≥ 18 years	46 (7%)	49.4 (11.7)	517 (18%)
NCT00106535	Roche	Tocilizumab	Placebo	3	1196	≥ 18 years	173 (14%)	52.0 (12.2)	989 (83%)
NCT01119859	Roche	Tocilizumab	Adalimumab	4	326	≥ 18 years	75 (23%)	53.9 (12.7)	262 (81%)
NCT01007435	Roche	Tocilizumab	Placebo	3	1162	≥ 18 years	180 (15%)	50.1 (13.5)	904 (78%)
NCT01232569	Roche	Tocilizumab	Placebo	3	656	≥ 18 years	81 (12%)	52.1 (11.5)	555 (85%)
COPD									
NCT01316913	GSK	Umeclidinium bromide	Tiotropium	3	872	≥ 40 years	455 (52%)	64.6 (8.4)	280 (32%)
NCT01316900	GSK	Umeclidinium bromide	Tiotropium	3	846	≥ 40 years	364 (43%)	62.9 (9.0)	261 (31%)
NCT01957163	GSK	Umeclidinium bromide	Fluticasone, Placebo	3	619	≥ 40 years	299 (48%)	64.4 (8.1)	212 (32%)
NCT02119286	GSK	Umeclidinium bromide	Fluticasone, Placebo	3	620	≥ 40 years	270 (44%)	62.9 (8.2)	228 (37%)

### Distribution of frailty

**Fig. 2:**
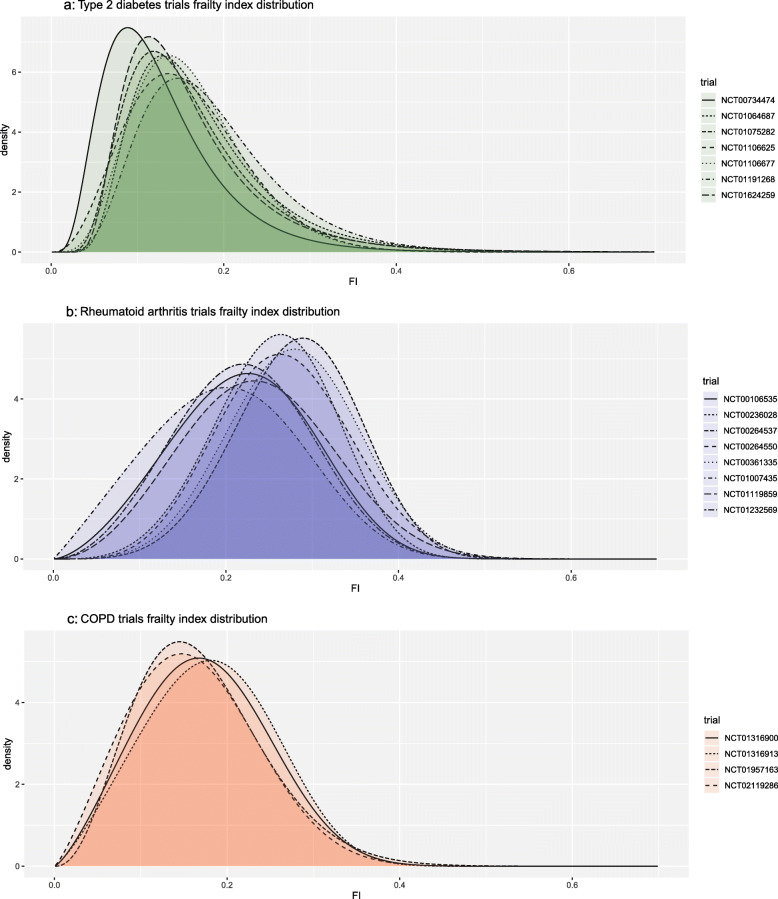
Distribution of frailty index in each trial. **a** Type 2 diabetes trials. **b** Rheumatoid arthritis trials. **c** COPD trials

The distribution of the frailty index for each trial is shown in Fig. 2. Each trial included participants with a wide range of frailty index values, and all trials included some frail participants. Distributions were similar within each index condition but differed substantially between the three conditions. Summary statistics for frailty in each trial, along with proportions in each category of frailty, are shown in Table 2. Taking an illustrative cut-off of 0.24 to indicate frailty, the proportion of trial participants with frailty ranged from 7 to 21% in T2DM trials, 33 to 73% in RA trials, and 15 to 22% in COPD trials.

**Table 2:** Mean frailty index, 99th centile, and proportion of participants in frailty categories for each trial: whole trial population and those over 65 years

Trial	Whole sample						Participants aged ≥ 65 years						Number missing (%)
	Mean frailty index	99th centile	Frailty index categories (%)				Mean frailty index	99th centile	Frailty index categories (%)				
			0–0.12	0.12–0.24	0.24–0.36	> 0.36			0–0.12	0.12–0.24	0.24–0.36	> 0.36	
Type 2 diabetes													
NCT01106625	0.16	0.35	31.36	56.12	11.82	0.7	0.17	0.51	24.38	63.68	8.81	3.13	74 (16%)
NCT01106677	0.16	0.35	28.26	59.79	11.21	0.74	0.17	0.33	20.13	70.29	9.08	0.5	124 (10%)
NCT00734474	0.13	0.35	54.22	39.24	5.72	0.82	0.14	0.32	43.78	49.08	6.81	0.33	66 (5%)
NCT01064687	0.17	0.45	26.9	56.56	13.44	3.1	0.19	0.46	20.49	57.86	17.54	4.1	4 (0.4%)
NCT01075282	0.16	0.42	33.35	53.02	11.29	2.34	0.18	0.42	23.2	57.15	16.72	2.93	4 (0.5%)
NCT01191268	0.18	0.43	20.73	58.29	17.82	3.16	0.2	0.42	14.96	60.54	21.21	3.29	2 (0.2%)
NCT01624259	0.16	0.45	35.34	51.53	10.36	2.77	0.18	0.43	24.7	55.16	16.94	3.2	1 (0.2%)
RA													
NCT00236028	0.26	0.41	2.62	37.05	53.73	6.6	0.27	0.41	1.15	28.08	61.5	9.27	12 (2%)
NCT00264537	0.28	0.44	1.14	25.99	58.83	14.05	0.31	0.46	0.16	15.63	63.43	20.78	4 (0.6%)
NCT00264550	0.27	0.45	2.03	34.71	51.37	11.89	0.28	0.41	3.58	24.91	57.11	14.41	2 (0.5%)
NCT00361335	0.28	0.45	1.49	29.24	55.43	13.85	0.29	0.46	0.73	26.31	56.99	15.96	1 (0.2%)
NCT00106535	0.22	0.4	11.57	46.97	37.19	4.27	0.24	0.4	9.73	39.92	44.71	5.64	4 (0.3%)
NCT01007435	0.2	0.4	18.92	47.79	29.95	3.35	0.21	0.41	15.89	46.55	32.81	4.75	9 (3%)
NCT01119859	0.24	0.44	8.5	43.27	39.88	8.36	0.25	0.48	7.07	40.41	40.3	12.22	3 (0.3%)
NCT01232569	0.22	0.4	10.32	49.31	36.44	3.93	0.23	0.4	7.59	47.37	41.24	3.8	0 (0%)
COPD													
NCT01316900	0.18	0.35	24.62	55.68	18.9	0.8	0.17	0.35	25.83	57.28	16.3	0.58	33 (4%)
NCT01316913	0.18	0.35	22.04	55.71	21.56	0.69	0.18	0.35	24.64	55.33	19.38	0.65	19 (2%)
NCT01957163	0.17	0.37	27.13	55.71	15.74	1.42	0.17	0.36	28.32	56.89	13.79	1	0 (0%)
NCT02119286	0.16	0.35	30.74	53.91	14.67	0.68	0.16	0.33	30.75	56.81	12.1	0.34	2 (0.3%)

### Relationship with baseline factors

**Fig. 3:**
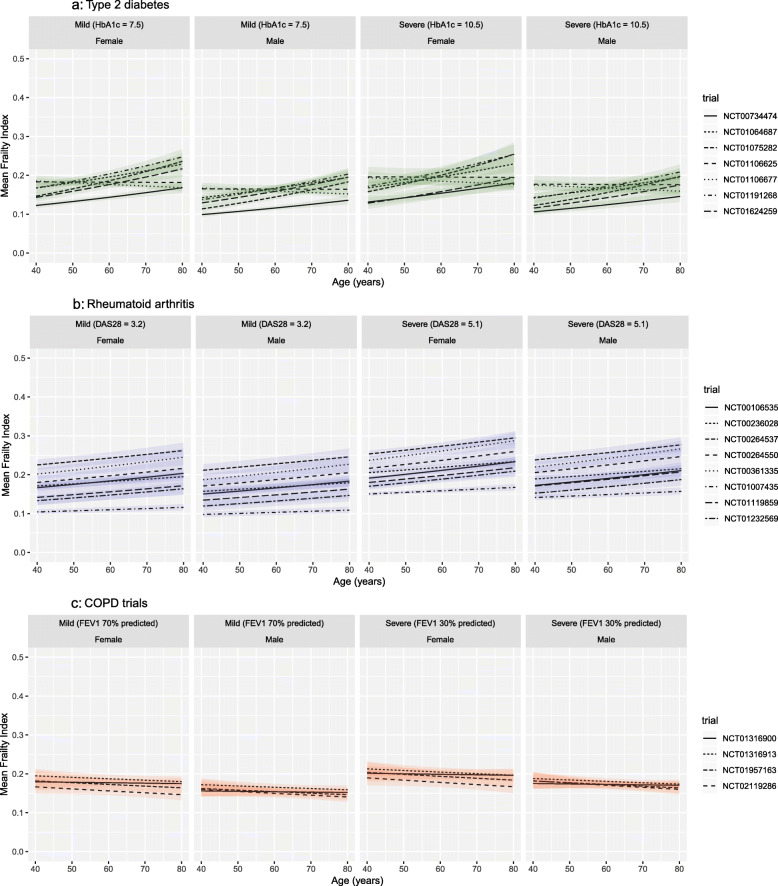
Relationship between age, sex, disease severity and frailty index. **a** Type 2 diabetes trials. **b** Rheumatoid arthritis trials. **c** COPD trials

Estimated mean frailty index by age, sex, and disease severity is shown in Fig. 3. Disease severity at baseline was associated with frailty index for COPD and, especially, for RA trials, but not for T2DM trials. Frailty was associated with female sex in all trials for all conditions. In the COPD trials, the mean frailty index was not associated with age, but for all of the RA trials and all but one T2DM trials, the mean frailty index increased with age. The variation by age was smaller than the variation between trials, however, and for all conditions, frailty remained common even among the youngest participants. For example, the modelled proportion of 40-year-olds with a frailty index > 0.24 ranged from 4 to 15%, 6 to 52%, and 20 to 27% in T2DM, RA, and COPD, respectively.

### Frailty index and serious adverse events

**Fig. 4:**
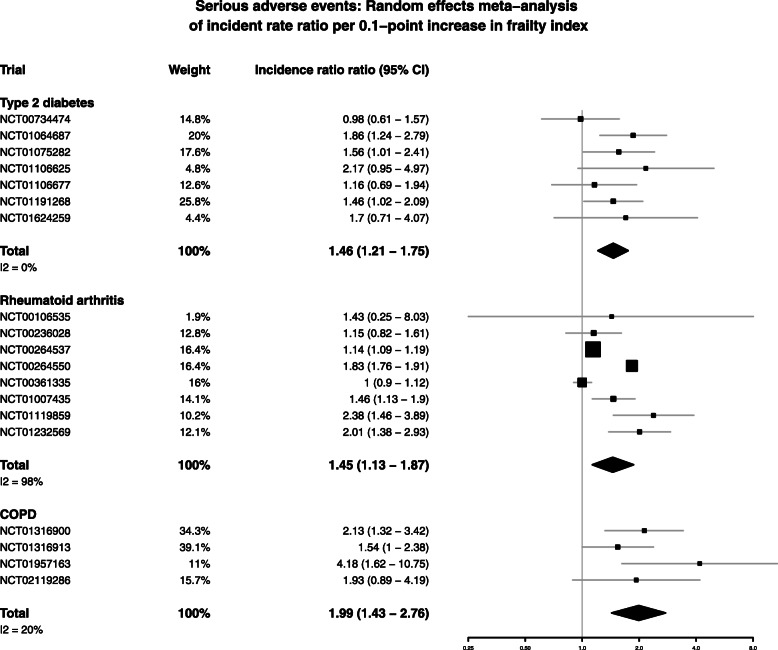
Random-effects meta-analysis of incidence rate ratio of serious adverse events per 0.1 point increase in frailty index. (**a**) Type 2 diabetes trials. (**b**) Rheumatoid arthritis trials. (**c**) COPD trials

The relationship between frailty index and the incidence of serious adverse events occurring during trial follow-up is summarised in Fig. 4. When the trials within each condition were meta-analysed, a 0.1-point increment in frailty index at baseline was associated with a higher serious adverse event rate for all conditions (IRR 1.46 (95% CI 1.21–1.75) for T2DM, 1.45 (1.13–1.87) for RA, and 1.99 (1.43–2.76) for COPD). Heterogeneity between trials was high for RA, but low for T2DM and COPD. The full model outputs for each trial are shown in the Supplementary appendix. Therefore, for each condition, after adjusting for age, sex, and disease severity, frailty index at baseline predicted subsequent serious adverse events.

## Discussion

### Summary of findings

Using individual-level participant data for 19 trials for three common and important chronic conditions—all with a mean age of less than 65 years—we found that frailty was highly prevalent among trial participants. The frailty index showed the expected relationships with sex, age (apart from in COPD), and disease severity and identified trial participants at higher risk of serious adverse events.

### In context of existing literature

Few studies have attempted to measure frailty across multiple clinical trials. To our knowledge, this is the first to include trials not specifically targeting older populations (with most participants aged < 65 years) and the first to do so for T2DM, RA, or COPD. Our findings that frailty can be identified in trials are consistent with two large hypertension trials, HYVET and SPRINT [10, 11], which focused on hypertension management in older people, and one heart failure trial in older people [12], which showed that frailty was relatively common in these trials. We extend these findings by showing that frailty is relatively common in ‘standard’ industry-funded phase 3 trials in younger populations, that it is associated with baseline characteristics, and that frailty at baseline predicts the risk of serious adverse events, even after adjusting for age, sex, and the severity of the index condition.

The frailty index in our analysis showed similar properties to observational studies of frailty using the frailty index approach [22, 23, 26]. As expected, the frailty index had a skewed distribution, was higher in women than men, and for RA and T2DM was associated with age. We have previously shown, using UK Biobank data, that frailty is identifiable in younger as well as older people [1], and the current work shows that this is also true of trials.

While many of the characteristics of the frailty index in the trial data are consistent with studies of frailty using observational cohorts and administrative data [28], the maximum frailty index in the trials (based on the 99th centile of the frailty index distribution) was lower than is typically seen in observational studies [29]. Since this difference was also evident among trial participants aged over 65, it cannot solely be attributed to the younger age of the trial participants. The extent to which this difference is due to trial eligibility criteria [15, 30] (e.g. comorbidities, renal function) or other selection pressures on trial participation (such as the need to be able to undergo multiple trial visits or procedures) is unknown. This suggests that our findings hold for the range of frailty index values we observed in these trials, which is narrower than that observed in unselected populations.

Importantly, while the very frailest patients were rarely included in the clinical trials, we found moderate to severely frail patients—who make up the bulk of those with frailty in the community—were commonly included as participants in clinical trials, despite those trials involving younger people aged under 65 years. Many trials require high disease activity/severity as inclusion criteria, which is one potential explanation for the high prevalence of frailty in some trials, particularly in conditions like RA where there is overlap between functional limitations resulting from active disease and deficits included in the frailty index.

It is notable that the frailty index in the COPD trials was not associated with increasing age, as would be expected. A similar phenomenon was also observed in both the SPRINT and TOPCAT trials (of hypertension and heart failure, respectively), whereby younger trial participants showed relatively higher frailty index values compared to relatively older trial participants [11, 12]. These COPD trials (as well as previous trials showing similar associations) may suggest that to be included in the trial, older people with COPD tended to be relatively less frail than similarly aged people with COPD in the general population. This could arise due to the trial selection process [31], as an example of collider bias, whereby conditioning on a subsequent outcome (trial inclusion) influences the relationship between causally proximal characteristics such as age and frailty [32]. We conducted exploratory analyses of the association between age and the St George Respiratory Questionnaire score and EQ 5D as these are known to increase with age in unselected populations [33, 34]. Like the frailty index, these were not associated with age in the COPD trials. Furthermore, the mean frailty index is lower in the COPD trials, and the range of frailty index values is narrower, compared to the frailty index distribution in previous observational studies of frailty in COPD [35]. This supports our speculation that the unexpected relationship between age and frailty index in these trials reflects differences between the trial population and people in the community with the same condition.

Frailty index was moderately associated with disease severity in COPD and RA. It would have been surprising had there been no association, as functional limitation and frailty, acting across multiple organ systems, are a well-recognised consequence of both diseases [19, 21]. Moreover, FEV1 has long been established as a marker of general physiological reserve as well as of lung disease. The fact that the correlation was not stronger is perhaps of greater interest as it suggests that factors other than the severity of the index disease are important drivers of frailty. Moreover, frailty index predicted adverse events independently of disease severity, indicating that the frailty index contains important clinical information about trial participants beyond that captured by disease-severity measures alone, possibly related to the increasing prevalence of multimorbidity.

### Strengths and limitations

A strength of our study is that we used a standard well-validated approach to measure frailty [26], across a large number of trials and a range of conditions, allowing comparison of findings between trials and between conditions. Our analysis also has some important limitations, however. The trials included were not a random sample, but instead were selected from trials that sponsors have made available to third-party researchers for secondary analyses. Not all sponsors share IPD, and those that do share data do not make all trials available. Of the trials we did access, not all trials had sufficient data to identify deficits for inclusion in a frailty index.

The data used to compile the frailty index were not collected for the purpose of identifying frailty, although this is true for most studies using the frailty index. Moreover, medical history data were redacted in most of the included trials, so we were therefore reliant on concomitant medication data to define long-term condition count-based deficits. Consequently, some conditions could only be included as part of a broader group (e.g. cardiovascular disease, obstructive airways disease) rather than as a specific condition, while other conditions (those without specific drug treatments) could not be included [16]. This restricts the number of conditions that could be included in our frailty index, and may result in an under-estimate of the number of conditions present (e.g. in people with multiple cardiovascular conditions which are counted as a single category, or with conditions such as chronic kidney disease which could not be identified using prescribed medications). Furthermore, we used existing instruments, primarily designed to characterise the index condition, to measure functional deficits of frailty (e.g. reduced mobility and difficulty with household tasks were identified using St George Respiratory Questionnaire in the context of COPD, and using the Health Assessment Questionnaire Disability Index in RA). It may be that instruments designed specifically to measure frailty would have improved sensitivity or specificity. Despite these limitations, and especially compared with most administrative data sources, trial data benefits from a wide range of physiological, biochemical, haematological, and functional measures. Moreover, given the regulatory conditions under which trials are conducted, these data were collected, recorded, and processed according to exacting standards.

### Implications

Current guidelines caution against the extrapolation of trial evidence to frail people [7, 20], and clinicians lack high-quality evidence about the benefits and harms of common treatments for people living with frailty. Our findings demonstrate that it is feasible to measure frailty, using an established, validated method—the frailty index—in standard industry-funded drug trials, and that on doing so significant numbers of trial participants have mild to moderate frailty. As such, while such trials cannot be claimed to be *representative* of people with frailty, particularly those with severe frailty who were very rarely found to be present, trials nonetheless contain important under-used information to help address current evidence gaps.

We were able to identify frailty in trials only because we were able to access trial IPD, which is complex and time-consuming. Moreover, several trials redacted data (and, less often, did not collect sufficient data) to allow us to calculate a frailty index. Both to allow clinicians to assess the degree to which frailty is under-represented in particular trials, and to understand whether and how treatment effects differ by frailty (realistically only feasible via meta-analysis of multiple trials), there is a need to expand existing trial conduct and reporting standards [36], to include standard measures of frailty. Our findings suggest that frailty is sufficiently common in trials for this to be a worthwhile exercise.

To that end, standard approaches to the collection and reporting of medical history data (to allow accurate assessment of comorbidities to be included in a frailty index) as well as measures specifically designed to assess frailty (e.g. the frailty phenotype) should be incorporated into international standards for the conduct of trials. Ideally, the adoption of complementary measures such as the frailty index and frailty phenotype measures should be considered. The frailty index can be applied to routinely collected trial data, but is likely to be more influenced by multimorbidity (and in turn, trial inclusion criteria) while the frailty phenotype may identify trial participants with more explicitly defined physiological frailty, some of whom may not have multimorbidity. Given the well-resourced and rigorous measurement and reporting usual in well-conducted trials, the adoption of standard measures of frailty across trials is highly feasible and would allow estimation of the impact of frailty on treatment effects both for individual trials and for meta-analyses of multiple trials. It would also enable identification of participants with increased frailty who are at increased risk of more serious adverse events, who might benefit from closer monitoring.

## Conclusion

Contrary to the prevailing view [7, 8], frailty, albeit not the most severe frailty, is common and readily measurable among clinical trial participants. This includes trials of relatively young populations. We have shown that participants with increased frailty at baseline also experience more serious adverse events, suggesting that such patients might merit closer monitoring and that screening for frailty should be considered for addition to future Consort checklists. Future research should evaluate whether frailty in trials is associated with treatment effectiveness. Both existing and future drug trials have the potential to inform the management of individuals living with frailty. Trialists therefore can and should routinely measure and report frailty. However, to do so frailty needs to become a standard measure within trials. Ideally, this would include both standardised assessment of comorbidities and baseline functional status (from which a frailty index could be consistently constructed) as well as physiological measurements to assess the frailty phenotype. There is also a need for research specifically targeting people with severe frailty, who were rarely included in these trials, and for whom the risks and benefits of treatments are most uncertain. Given ageing population demographics as well as the presence of frailty among relatively younger people, such measures would be central to understanding how treatments should be applied to the growing numbers of people living with frailty.

## Supplementary information

**Additional file 1: **Deficits included in frailty index for each condition. **Table S1**: Diabetes trials frailty index deficits. **Table S2**: Rheumatoid arthritis trials frailty index deficits. **Table S3**: COPD trials frailty index deficits. Parameters for the distributions of the frailty index for each trial. **Table S4**: Parameters of generalised gamma distribution for frailty index for each trial. Generalised gamma model coefficients and variance covariance matrices. **Tables S5-S42**: Coefficients and variance-covariance matrices for generalised gamma models assessing relationship between frailty index and baseline characteristics for each trial.

## Data Availability

The data that support the findings of this study are available from Clinical Study Data Request and the Yale Open Data Access repositories but restrictions apply to the availability of these data, which were used under license for the current study, and so are not publicly available. Data are however available by directly applying to these repositories via application process to their respective independent data access committees. All data released from the respective safe havens (Clinical Study Data Request and the Yale Open Data Access) has been made available via the Supplementary appendix.
