# Supplementary material for: Identifying frailty in trials: an analysis of individual participant data from trials of novel pharmacological interventions
Source: BMC Med. 2020 Oct 22;18:309. doi: 10.1186/s12916-020-01752-1 (PMC7579922; doi:10.1186/s12916-020-01752-1)
Supplement: Supplementary file 1 — Additional file 1: Deficits included in frailty index for each condition. Table S1: Diabetes trials frailty index deficits. Table S2: Rheumatoid arthritis trials frailty index deficits. Table S3: COPD trials frailty index deficits. Parameters for the distributions of the frailty index for each trial. Table S4: Parameters of generalised gamma distribution for frailty index for each trial. Generalised gamma model coefficients and variance covariance matrices. Tables S5-S42: Coefficients and variance-covariance matrices for generalised gamma models assessing relationship between frailty index and baseline characteristics for each trial. [file 12916_2020_1752_MOESM1_ESM.docx]

Identifying frailty in trials: an analysis of individual participant data from trials of novel pharmacological interventions

Supplementary appendix

Peter Hanlon

Elaine Butterly

Jim Lewsey

Stefan Siebert

Frances S Mair

David A McAllister

Table of Contents

[Deficits included in frailty index for each condition 2](#_Toc42070486)

[Parameters for the distributions of the frailty index for each trial 7](#_Toc42070487)

[Generalised gamma model coefficients and variance covariance matrices 8](#_Toc42070488)

# Deficits included in frailty index for each condition

| **Table S1: Diabetes trials frailty index deficits** | | |
| --- | --- | --- |
| **Deficit** | **Source** | **Coding** |
| Acid-related disorders | Concomitant medications | Present = 1, absent = 0 |
| Diabetes mellitus | Concomitant medications | Present = 1, absent = 0 |
| Thromboemolic disease/AF | Concomitant medications | Present = 1, absent = 0 |
| Cardiovascular disease | Concomitant medications | Present = 1, absent = 0 |
| Urinary tract disorder/incontinence | Concomitant medications | Present = 1, absent = 0 |
| Glaucoma | Concomitant medications | Present = 1, absent = 0 |
| Arthritis and arthralgia | Concomitant medications | Present = 1, absent = 0 |
| Osteoporosis | Concomitant medications | Present = 1, absent = 0 |
| Gout | Concomitant medications | Present = 1, absent = 0 |
| Inflammatory conditions (arthopathies, IBD, connective tissue diseases) | Concomitant medications | Present = 1, absent = 0 |
| Migraine | Concomitant medications | Present = 1, absent = 0 |
| Chronic pain | Concomitant medications | Present = 1, absent = 0 |
| Schizophrenia and delusional dosirders | Concomitant medications | Present = 1, absent = 0 |
| Affective disorders/sleep disorders | Concomitant medications | Present = 1, absent = 0 |
| Epilepsy | Concomitant medications | Present = 1, absent = 0 |
| Parkinson's disease/parksinsonism | Concomitant medications | Present = 1, absent = 0 |
| Dementia | Concomitant medications | Present = 1, absent = 0 |
| Chronic lower respiratory disease | Concomitant medications | Present = 1, absent = 0 |
| Thyroid disorders | Concomitant medications | Present = 1, absent = 0 |
| Skin disorders | Concomitant medications | Present = 1, absent = 0 |
| Difficulty picking up objects | IWQOL1 | Present = 1, absent = 0 |
| Difficulty getting up from chairs | IWQOL3 | Present = 1, absent = 0 |
| Trouble with stairs | IWQOL4 | Present = 1, absent = 0 |
| Difficulty dressing | IWQOL5 | Present = 1, absent = 0 |
| Difficulty with mobility | IWQOL6 | Present = 1, absent = 0 |
| Short of breath on mild exertion | IWQOL8 | Present = 1, absent = 0 |
| Self-rated health | EQ5D/SF36-1 | ((Total out of 100)-100)/100 |
| Limited mobility/difficulty walking several blocks | EQ5D-1/SF36-10 | Severe difficulty/unable = 1, some difficulty = 0.5, no difficulty = 0 |
| Difficulty with self-care | EQ5D-2/SF36-12 | Severe difficulty/unable = 1, some difficulty = 0.5, no difficulty = 0 |
| Limited in usual activities | EQ5D-3/SF25-32 | Severe difficulty/unable = 1, some difficulty = 0.5, no difficulty = 0 |
| Pain | EQ5D-4/SF36-21 | Severe difficulty/unable = 1, some difficulty = 0.5, no difficulty = 0 |
| Anxiety or Down in dumps | EQ5D-5/SF36-25 | Severe difficulty/unable = 1, some difficulty = 0.5, no difficulty = 0 |
| eGFR | baseline laboratory measures | <30 = 1, <60 = 0.5, >60 = 0 |
| Haemoblobin | baseline laboratory measures | <115 = 1 (men), <110 = 1 (women) |
| Fib4 | baseline laboratory measures | >2.67 = 1, >2 = 0.5, <2 = 0 |
| Sodium | baseline laboratory measures | <133 = 1 |
| Calcium | baseline laboratory measures | >2.7 mmol/L = 1, <2.7 = 0 |
| Cholesterol | baseline laboratory measures | >6.2 mmol/L = 1, <6.2 = 0 |
| Systolic blood pressure | baseline assessment | >150 = 1 |
| Body mass index | baseline assessment | <18.5 or >30 = 1, >25 = 0.5, 18.5-25 = 0 |

| **Table S2: Rheumatoid arthritis trials frailty index deficits** | | |
| --- | --- | --- |
| **Deficit** | **Source** | **Coding** |
| Acid-related disorders | Concomitant medications | Present = 1, absent = 0 |
| Diabetes mellitus | Concomitant medications | Present = 1, absent = 0 |
| Thromboemolic disease/AF | Concomitant medications | Present = 1, absent = 0 |
| Cardiovascular disease | Concomitant medications | Present = 1, absent = 0 |
| Urinary tract disorder/incontinence | Concomitant medications | Present = 1, absent = 0 |
| Glaucoma | Concomitant medications | Present = 1, absent = 0 |
| Osteoporosis | Concomitant medications | Present = 1, absent = 0 |
| Gout | Concomitant medications | Present = 1, absent = 0 |
| Inflammatory conditions (arthopathies, IBD, connective tissue diseases) | Concomitant medications | Present = 1, absent = 0 |
| Migraine | Concomitant medications | Present = 1, absent = 0 |
| Chronic pain | Concomitant medications | Present = 1, absent = 0 |
| Schizophrenia and delusional dosirders | Concomitant medications | Present = 1, absent = 0 |
| Affective disorders/sleep disorders | Concomitant medications | Present = 1, absent = 0 |
| Epilepsy | Concomitant medications | Present = 1, absent = 0 |
| Parkinson's disease/parksinsonism | Concomitant medications | Present = 1, absent = 0 |
| Dementia | Concomitant medications | Present = 1, absent = 0 |
| Chronic lower respiratory disease | Concomitant medications | Present = 1, absent = 0 |
| Thyroid disorders | Concomitant medications | Present = 1, absent = 0 |
| Skin disorders | Concomitant medications | Present = 1, absent = 0 |
| Difficulty getting out of bed | HAQ-DI | Severe difficulty/unable = 1, some difficulty = 0.5, no difficulty = 0 |
| Difficulty with household chores | HAQ-DI | Severe difficulty/unable = 1, some difficulty = 0.5, no difficulty = 0 |
| Difficulty climbing stairs | HAQ-DI | Severe difficulty/unable = 1, some difficulty = 0.5, no difficulty = 0 |
| Difficulty with shopping (groceries) | HAQ-DI | Severe difficulty/unable = 1, some difficulty = 0.5, no difficulty = 0 |
| Difficult standing | HAQ-DI | Severe difficulty/unable = 1, some difficulty = 0.5, no difficulty = 0 |
| Difficulty with toilet | HAQ-DI | Severe difficulty/unable = 1, some difficulty = 0.5, no difficulty = 0 |
| Self-rated health | EQ5D/SF36-1 | ((Total out of 100)-100)/100 |
| Limited mobility | EQ5D-1/SF36-10 | Severe difficulty/unable = 1, some difficulty = 0.5, no difficulty = 0 |
| Difficulty with self-care | EQ5D-2/SF36-12 | Severe difficulty/unable = 1, some difficulty = 0.5, no difficulty = 0 |
| Limited in usual activities | EQ5D-3/SF25-32 | Severe difficulty/unable = 1, some difficulty = 0.5, no difficulty = 0 |
| Pain | EQ5D-4/SF36-21 | Severe difficulty/unable = 1, some difficulty = 0.5, no difficulty = 0 |
| Anxiety | EQ5D-5/SF36-25 | Severe difficulty/unable = 1, some difficulty = 0.5, no difficulty = 0 |
| eGFR | baseline laboratory measures | <30 = 1, <60 = 0.5, >60 = 0 |
| Haemoblobin | baseline laboratory measures | <115 = 1 (men), <110 = 1 (women) |
| Fib4 | baseline laboratory measures | >2.67 = 1, >2 = 0.5, <2 = 0 |
| Sodium | baseline laboratory measures | <133 = 1 |
| Calcium | baseline laboratory measures | >2.7 mmol/L = 1, <2.7 = 0 |
| Glucose | baseline laboratory measures | >11 mmol/L = 1, >7 = 0.5, <7 = 0 |
| Cholesterol | baseline laboratory measures | >6.2 mmol/L = 1, <6.2 = 0 |
| Systolic blood pressure | baseline assessment | >150 = 1 |
| Body mass index | baseline assessment | <18.5 or >30 = 1, >25 = 0.5, 18.5-25 = 0 |

| **Table S3: COPD trials frailty index deficits** | | |
| --- | --- | --- |
| **Deficit** | **Source** | **Coding** |
| Acid-related disorders | Concomitant medications | Present = 1, absent = 0 |
| Diabetes mellitus | Concomitant medications | Present = 1, absent = 0 |
| Thromboemolic disease/AF | Concomitant medications | Present = 1, absent = 0 |
| Cardiovascular disease | Concomitant medications | Present = 1, absent = 0 |
| Urinary tract disorder/incontinence | Concomitant medications | Present = 1, absent = 0 |
| Glaucoma | Concomitant medications | Present = 1, absent = 0 |
| Arthritis and arthralgia | Concomitant medications | Present = 1, absent = 0 |
| Osteoporosis | Concomitant medications | Present = 1, absent = 0 |
| Gout | Concomitant medications | Present = 1, absent = 0 |
| Inflammatory conditions (arthopathies, IBD, connective tissue diseases) | Concomitant medications | Present = 1, absent = 0 |
| Migraine | Concomitant medications | Present = 1, absent = 0 |
| Chronic pain | Concomitant medications | Present = 1, absent = 0 |
| Schizophrenia and delusional dosirders | Concomitant medications | Present = 1, absent = 0 |
| Affective disorders/sleep disorders | Concomitant medications | Present = 1, absent = 0 |
| Epilepsy | Concomitant medications | Present = 1, absent = 0 |
| Parkinson's disease/parksinsonism | Concomitant medications | Present = 1, absent = 0 |
| Dementia | Concomitant medications | Present = 1, absent = 0 |
| Chronic lower respiratory disease | Concomitant medications | Present = 1, absent = 0 |
| Thyroid disorders | Concomitant medications | Present = 1, absent = 0 |
| Skin disorders | Concomitant medications | Present = 1, absent = 0 |
| Difficulty with Stairs | SGRQ | Present = 1, absent = 0 |
| Difficulty with Dressing | SGRQ | Present = 1, absent = 0 |
| Difficulty with Housework | SGRQ | Present = 1, absent = 0 |
| Difficulty with Shopping | SGRQ | Present = 1, absent = 0 |
| Difficulty with Sports | SGRQ | Present = 1, absent = 0 |
| Bath/shower long time | SGRQ | Present = 1, absent = 0 |
| Everything too much effort | SGRQ | Present = 1, absent = 0 |
| Feel that exercise not safe for me | SGRQ | Present = 1, absent = 0 |
| Feel frail because of chest | SGRQ | Present = 1, absent = 0 |
| Panic | SGRQ | Present = 1, absent = 0 |
| Exhausted easily | SGRQ | Present = 1, absent = 0 |
| eGFR | baseline laboratory measures | <30 = 1, <60 = 0.5, >60 = 0 |
| Haemoglobin | baseline laboratory measures | <115 = 1 (men), <110 = 1 (women) |
| Fib4 | baseline laboratory measures | >2.67 = 1, >2 = 0.5, <2 = 0 |
| Sodium | baseline laboratory measures | <133 = 1 |
| Calcium | baseline laboratory measures | >2.7 mmol/L = 1, <2.7 = 0 |
| Glucose | baseline laboratory measures | >11 mmol/L = 1, >7 = 0.5, <7 = 0 |
| Systolic BP | baseline assessment | >150 = 1 |
| Body mass index | baseline assessment | <18.5 or >30 = 1, >25 = 0.5, 18.5-25 = 0 |

Parameters for the distributions of the frailty index for each trial

Table S4: Generalised gamma distribution. P-value for fit (Kolmogorov Smirnov test) – p>0.05 indicated good fit.

| Trial | Mu | Sigma | Q | P-value |
| --- | --- | --- | --- | --- |
| NCT00734474 | -2.1384099 | 0.5184630 | 0.2126836 | 0.6536614 |
| NCT01064687 | -1.8686473 | 0.4334530 | -0.0911457 | 0.3841132 |
| NCT01075282 | -1.9262997 | 0.4534190 | -0.0070541 | 0.1537916 |
| NCT01191268 | -1.7281470 | 0.4233779 | 0.2312597 | 0.1205054 |
| NCT01624259 | -1.9900970 | 0.4453075 | -0.2423449 | 0.3977397 |
| NCT01106625 | -1.7845810 | 0.4249601 | 0.6854553 | 0.7535057 |
| NCT01106677 | -1.8141929 | 0.4008267 | 0.4329018 | 0.0994761 |
| NCT00106535 | -1.3622950 | 0.3178938 | 1.2024470 | 0.1125004 |
| NCT01007435 | -1.4211607 | 0.3644020 | 1.3289993 | 0.1304014 |
| NCT01119859 | -1.3239383 | 0.3311765 | 0.9893788 | 0.6863576 |
| NCT01232569 | -1.3903390 | 0.3191592 | 1.0485768 | 0.5766842 |
| NCT00236028 | -1.2671025 | 0.2418719 | 0.9600227 | 0.2527657 |
| NCT00264537 | -1.1828921 | 0.2272744 | 0.8935029 | 0.7031388 |
| NCT00264550 | -1.2542247 | 0.2725728 | 0.7223240 | 0.0897029 |
| NCT00361335 | -1.2060284 | 0.2486708 | 0.8211526 | 0.4014494 |
| NCT01316900 | -1.5972347 | 0.3817339 | 1.1277266 | 0.1655973 |
| NCT01316913 | -1.5434427 | 0.3536005 | 1.2548760 | 0.0574210 |
| NCT01957163 | -1.7122818 | 0.4290410 | 0.7032241 | 0.0350085 |
| NCT02119286 | -1.6826762 | 0.4197403 | 1.0602738 | 0.2451722 |

Generalised gamma model coefficients and variance covariance matrices

Each of these describes a generalised gamma model for FI values on age (centred at 60 years), sex (male = 1, female = 0) and disease severity

| Table S5: NCT00734474: generalised gamma model coefficients | |
| --- | --- |
| Mu | -1.99992 |
| Sigma | -0.70364 |
| Q | 0.203893 |
| Age | 0.007928 |
| Sex | -0.21326 |
| HbA1c | 0.023292 |

| Table S6: NCT00734474: variance covariance matrix from generalised gamma model | | | | | | |
| --- | --- | --- | --- | --- | --- | --- |
|  | Mu | sigma | Q | Age (centred) | Sex | HbA1c |
| Mu | 0.000813 | -9.8E-05 | 0.001435 | 1.13E-05 | -0.00039 | -4.4E-05 |
| Sigma | -9.8E-05 | 0.000439 | -0.00031 | 3E-07 | 4E-07 | 3.9E-06 |
| Q | 0.001435 | -0.00031 | 0.005856 | -4.9E-06 | -5.8E-06 | -5.7E-05 |
| Age | 1.13E-05 | 3E-07 | -4.9E-06 | 2.1E-06 | 0 | -2E-07 |
| Sex | -0.00039 | 4E-07 | -5.8E-06 | 0 | 0.000823 | 1.31E-05 |
| HbA1c | -4.4E-05 | 3.9E-06 | -5.7E-05 | -2E-07 | 1.31E-05 | 0.000176 |

| Table S7: NCT01064687: generalised gamma model coefficients | |
| --- | --- |
| Mu | -1.73457 |
| Sigma | -0.86705 |
| Q | -0.0698 |
| Age | 0.007821 |
| Sex | -0.16049 |
| HbA1c | 0.000813 |

| Table S8: NCT01064687: variance covariance matrix from generalised gamma model | | | | | | |
| --- | --- | --- | --- | --- | --- | --- |
|  | Mu | sigma | Q | Age (centred) | Sex | HbA1c |
| Mu | 0.000826 | 3.61E-05 | 0.001552 | 8.5E-06 | -0.00049 | 1.77E-05 |
| Sigma | 3.61E-05 | 0.000514 | 0.000127 | -1E-07 | -4E-06 | 6E-07 |
| Q | 0.001552 | 0.000127 | 0.006997 | -3.9E-06 | -0.00017 | 2.45E-05 |
| Age | 8.5E-06 | -1E-07 | -3.9E-06 | 1.9E-06 | -1.9E-06 | 1E-07 |
| Sex | -0.00049 | -4E-06 | -0.00017 | -1.9E-06 | 0.00076 | -3.5E-05 |
| HbA1c | 1.77E-05 | 6E-07 | 2.45E-05 | 1E-07 | -3.5E-05 | 0.000106 |

| Table S9: NCT01075282: generalised gamma model coefficients | |
| --- | --- |
| Mu | -1.74062 |
| Sigma | -0.86522 |
| Q | 0.080101 |
| Age | 0.011933 |
| Sex | -0.25352 |
| HbA1c | 0.024706 |

| Table S10: NCT01075282: variance covariance matrix from generalised gamma model | | | | | | |
| --- | --- | --- | --- | --- | --- | --- |
|  | Mu | sigma | Q | Age (centred) | Sex | HbA1c |
| Mu | 0.00093 | -5.3E-05 | 0.002002 | 8.1E-06 | -0.00051 | -4.3E-05 |
| Sigma | -5.3E-05 | 0.000608 | -0.00019 | 2E-07 | 8.4E-06 | 0.000002 |
| Q | 0.002002 | -0.00019 | 0.008797 | -6.1E-06 | -0.00031 | -7.6E-05 |
| Age | 8.1E-06 | 2E-07 | -6.1E-06 | 2.4E-06 | -2.2E-06 | 3E-07 |
| Sex | -0.00051 | 8.4E-06 | -0.00031 | -2.2E-06 | 0.000869 | -4.6E-06 |
| HbA1c | -4.3E-05 | 0.000002 | -7.6E-05 | 3E-07 | -4.6E-06 | 0.000217 |

| Table S11: NCT01191268: generalised gamma model coefficients | |
| --- | --- |
| Mu | -1.63563 |
| Sigma | -0.9037 |
| Q | 0.163278 |
| Age | 0.009778 |
| Sex | -0.19765 |
| HbA1c | 0.008889 |

| Table S12: NCT01191268: variance covariance matrix from generalised gamma model | | | | | | |
| --- | --- | --- | --- | --- | --- | --- |
|  | Mu | sigma | Q | Age (centred) | Sex | HbA1c |
| Mu | 0.000747 | -8.7E-05 | 0.001595 | -2.2E-06 | -0.00037 | -9E-05 |
| Sigma | -8.7E-05 | 0.00058 | -0.00035 | 0.000001 | -6.9E-06 | 2.7E-06 |
| Q | 0.001595 | -0.00035 | 0.008136 | -1.9E-05 | 0.000126 | -5E-05 |
| Age | -2.2E-06 | 0.000001 | -1.9E-05 | 2.3E-06 | -2E-06 | 0.000002 |
| Sex | -0.00037 | -6.9E-06 | 0.000126 | -2E-06 | 0.000743 | 4.9E-06 |
| HbA1c | -9E-05 | 2.7E-06 | -5E-05 | 0.000002 | 4.9E-06 | 0.00017 |

| Table S13: NCT01624259: generalised gamma model coefficients | |
| --- | --- |
| Mu | -1.87022 |
| Sigma | -0.83679 |
| Q | -0.09601 |
| Age | 0.01037 |
| Sex | -0.10408 |
| HbA1c | -0.03629 |

| Table S14: NCT01624259: variance covariance matrix from generalised gamma model | | | | | | |
| --- | --- | --- | --- | --- | --- | --- |
|  | Mu | sigma | Q | Age (centred) | Sex | HbA1c |
| Mu | 0.001502 | 0.000112 | 0.003486 | 2.07E-05 | -0.00075 | -4.4E-05 |
| Sigma | 0.000112 | 0.000847 | 0.000372 | 1.2E-06 | -1.9E-05 | -3.5E-06 |
| Q | 0.003486 | 0.000372 | 0.014139 | 3.75E-05 | -0.0006 | -0.00011 |
| Age | 2.07E-05 | 1.2E-06 | 3.75E-05 | 3.5E-06 | -2.2E-06 | 5.5E-06 |
| Sex | -0.00075 | -1.9E-05 | -0.0006 | -2.2E-06 | 0.001282 | 2.58E-05 |
| HbA1c | -4.4E-05 | -3.5E-06 | -0.00011 | 5.5E-06 | 2.58E-05 | 0.000502 |

| Table S15: NCT01106625: generalised gamma model coefficients | |
| --- | --- |
| Mu | -1.80232 |
| Sigma | -1.1759 |
| Q | -0.37176 |
| Age | -0.00021 |
| Sex | -0.1019 |
| HbA1c | 0.023065 |

| Table S16: NCT01106625: variance covariance matrix from generalised gamma model | | | | | | |
| --- | --- | --- | --- | --- | --- | --- |
|  | Mu | sigma | Q | Age (centred) | Sex | HbA1c |
| Mu | 0.001582 | 0.000589 | 0.004772 | 0.000024 | -0.00083 | -1.9E-05 |
| Sigma | 0.000589 | 0.00194 | 0.002561 | 0.000007 | -0.00013 | -1.7E-05 |
| Q | 0.004772 | 0.002561 | 0.025632 | 5.71E-05 | -0.00103 | -0.00014 |
| Age | 0.000024 | 0.000007 | 5.71E-05 | 3.6E-06 | -3.3E-06 | 3.1E-06 |
| Sex | -0.00083 | -0.00013 | -0.00103 | -3.3E-06 | 0.001296 | -7.4E-05 |
| HbA1c | -1.9E-05 | -1.7E-05 | -0.00014 | 3.1E-06 | -7.4E-05 | 0.000379 |

| Table S17: NCT01106677: generalised gamma model coefficients | |
| --- | --- |
| Mu | -1.84125 |
| Sigma | -1.12867 |
| Q | -0.34734 |
| Age | -0.00236 |
| Sex | -0.10274 |
| HbA1c | 0.015572 |

| Table S18: NCT01106677: variance covariance matrix from generalised gamma model | | | | | | |
| --- | --- | --- | --- | --- | --- | --- |
|  | Mu | sigma | Q | Age (centred) | Sex | HbA1c |
| Mu | 0.000447 | 0.000154 | 0.001337 | 5.9E-06 | -0.00021 | 1.56E-05 |
| Sigma | 0.000154 | 0.000584 | 0.000738 | 4E-07 | -8.4E-06 | -5.2E-06 |
| Q | 0.001337 | 0.000738 | 0.007892 | 3.3E-06 | -7.3E-05 | -4.5E-05 |
| Age | 5.9E-06 | 4E-07 | 3.3E-06 | 1.2E-06 | -1E-07 | 1.1E-06 |
| Sex | -0.00021 | -8.4E-06 | -7.3E-05 | -1E-07 | 0.000425 | -2.1E-05 |
| HbA1c | 1.56E-05 | -5.2E-06 | -4.5E-05 | 1.1E-06 | -2.1E-05 | 0.000134 |

| Table S19: NCT00106535: generalised gamma model coefficients | |
| --- | --- |
| Mu | -1.7998 |
| Sigma | -1.17989 |
| Q | 1.089106 |
| Age | 0.004992 |
| Sex | -0.10346 |
| DAS-28 | 0.072516 |

| Table S20: NCT00106535: variance covariance matrix from generalised gamma model | | | | | | |
| --- | --- | --- | --- | --- | --- | --- |
|  | Mu | sigma | Q | Age (centred) | Sex | DAS-28 |
| Mu | 0.002681 | -0.00057 | 0.001884 | 2.3E-06 | -0.00017 | -0.00035 |
| Sigma | -0.00057 | 0.000838 | -0.00132 | 2.3E-06 | -4.7E-05 | 4.99E-05 |
| Q | 0.001884 | -0.00132 | 0.005923 | -7.5E-06 | 0.000156 | -0.00016 |
| Age | 2.3E-06 | 2.3E-06 | -7.5E-06 | 6E-07 | 6E-07 | 2E-07 |
| Sex | -0.00017 | -4.7E-05 | 0.000156 | 6E-07 | 0.000606 | 1.64E-05 |
| DAS-28 | -0.00035 | 4.99E-05 | -0.00016 | 2E-07 | 1.64E-05 | 5.11E-05 |

| Table S21: NCT01007435: generalised gamma model coefficients | |
| --- | --- |
| Mu | -2.74094 |
| Sigma | -1.05458 |
| Q | 0.839224 |
| Age | 0.002688 |
| Sex | -0.06333 |
| DAS-28 | 0.193328 |

| Table S22: NCT01007435: variance covariance matrix from generalised gamma model | | | | | | |
| --- | --- | --- | --- | --- | --- | --- |
|  | Mu | sigma | Q | Age (centred) | Sex | DAS-28 |
| Mu | 0.004236 | -0.00081 | 0.003118 | 1.6E-06 | -4.6E-05 | -0.00056 |
| Sigma | -0.00081 | 0.000639 | -0.00109 | 1.9E-06 | -2.4E-05 | 9.16E-05 |
| Q | 0.003118 | -0.00109 | 0.00537 | -7.2E-06 | 9.28E-05 | -0.00035 |
| Age | 1.6E-06 | 1.9E-06 | -7.2E-06 | 5E-07 | -8E-07 | 4E-07 |
| Sex | -4.6E-05 | -2.4E-05 | 9.28E-05 | -8E-07 | 0.000529 | -8.8E-06 |
| DAS-28 | -0.00056 | 9.16E-05 | -0.00035 | 4E-07 | -8.8E-06 | 0.000079 |

| Table S23: NCT01119859: generalised gamma model coefficients | |
| --- | --- |
| Mu | -2.15833 |
| Sigma | -1.18086 |
| Q | 0.958357 |
| Age | 0.004792 |
| Sex | -0.05208 |
| DAS-28 | 0.125889 |

| Table S24: NCT01119859: variance covariance matrix from generalised gamma model | | | | | | |
| --- | --- | --- | --- | --- | --- | --- |
|  | Mu | sigma | Q | Age (centred) | Sex | DAS-28 |
| Mu | 0.018213 | -0.00252 | 0.008921 | -1.2E-05 | -0.00068 | -0.00243 |
| Sigma | -0.00252 | 0.003155 | -0.00572 | 8E-07 | -0.00028 | 0.000212 |
| Q | 0.008921 | -0.00572 | 0.025457 | -2.7E-06 | 0.000997 | -0.00075 |
| Age | -1.2E-05 | 8E-07 | -2.7E-06 | 1.9E-06 | -6.3E-06 | 3.6E-06 |
| Sex | -0.00068 | -0.00028 | 0.000997 | -6.3E-06 | 0.001953 | 6.36E-05 |
| DAS-28 | -0.00243 | 0.000212 | -0.00075 | 3.6E-06 | 6.36E-05 | 0.000343 |

| Table S25: NCT01232569: generalised gamma model coefficients | |
| --- | --- |
| Mu | -2.22043 |
| Sigma | -1.23044 |
| Q | 0.982978 |
| Age | 0.005164 |
| Sex | -0.11025 |
| DAS-28 | 0.128846 |

| Table S26: NCT01232569: variance covariance matrix from generalised gamma model | | | | | | |
| --- | --- | --- | --- | --- | --- | --- |
|  | Mu | sigma | Q | Age (centred) | Sex | DAS-28 |
| Mu | 0.007379 | -0.00117 | 0.004063 | -2.4E-06 | 7.23E-05 | -0.00099 |
| Sigma | -0.00117 | 0.001415 | -0.00225 | 5.4E-06 | -0.00006 | 0.000115 |
| Q | 0.004063 | -0.00225 | 0.010483 | -1.9E-05 | 0.000209 | -0.0004 |
| Age | -2.4E-06 | 5.4E-06 | -1.9E-05 | 1.2E-06 | -2.6E-06 | 1.4E-06 |
| Sex | 7.23E-05 | -0.00006 | 0.000209 | -2.6E-06 | 0.001021 | -3.2E-05 |
| DAS-28 | -0.00099 | 0.000115 | -0.0004 | 1.4E-06 | -3.2E-05 | 0.000142 |

| Table S27: NCT00236028: generalised gamma model coefficients | |
| --- | --- |
| Mu | -1.94368 |
| Sigma | -1.51325 |
| Q | 0.804074 |
| Age | 0.00327 |
| Sex | -0.0831 |
| DAS-28 | 0.096618 |

| Table S28: NCT00236028: variance covariance matrix from generalised gamma model | | | | | | |
| --- | --- | --- | --- | --- | --- | --- |
|  | Mu | sigma | Q | Age (centred) | Sex | DAS-28 |
| Mu | 0.002321 | -0.00046 | 0.001825 | -9E-07 | -2.5E-05 | -0.00028 |
| Sigma | -0.00046 | 0.000828 | -0.0013 | 1.6E-06 | -3.3E-05 | 3.97E-05 |
| Q | 0.001825 | -0.0013 | 0.006823 | -6.5E-06 | 0.000132 | -0.00016 |
| Age | -9E-07 | 1.6E-06 | -6.5E-06 | 3E-07 | -1.4E-06 | 5E-07 |
| Sex | -2.5E-05 | -3.3E-05 | 0.000132 | -1.4E-06 | 0.000246 | -6E-06 |
| DAS-28 | -0.00028 | 3.97E-05 | -0.00016 | 5E-07 | -6E-06 | 3.75E-05 |

| Table S29: NCT00264537: generalised gamma model coefficients | |
| --- | --- |
| Mu | -1.57697 |
| Sigma | -1.51704 |
| Q | 0.556751 |
| Age | 0.003795 |
| Sex | -0.06288 |
| DAS-28 | 0.062217 |

| Table S30: NCT00264537: variance covariance matrix from generalised gamma model | | | | | | |
| --- | --- | --- | --- | --- | --- | --- |
|  | Mu | sigma | Q | Age (centred) | Sex | DAS-28 |
| Mu | 0.003785 | -0.00067 | 0.003663 | 4.2E-06 | -0.00011 | -0.00049 |
| Sigma | -0.00067 | 0.00129 | -0.00189 | 3.3E-06 | -3.9E-05 | 0.000066 |
| Q | 0.003663 | -0.00189 | 0.013283 | -1.8E-05 | 0.000212 | -0.00036 |
| Age | 4.2E-06 | 3.3E-06 | -1.8E-05 | 6E-07 | -1E-06 | 0 |
| Sex | -0.00011 | -3.9E-05 | 0.000212 | -1E-06 | 0.000638 | 1.8E-06 |
| DAS-28 | -0.00049 | 0.000066 | -0.00036 | 0 | 1.8E-06 | 6.75E-05 |

| Table S31: NCT00264550: generalised gamma model coefficients | |
| --- | --- |
| Mu | -1.89756 |
| Sigma | -1.39654 |
| Q | 0.526478 |
| Age | 0.004579 |
| Sex | -0.05166 |
| DAS-28 | 0.096635 |

| Table S32: NCT00264550: variance covariance matrix from generalised gamma model | | | | | | |
| --- | --- | --- | --- | --- | --- | --- |
|  | Mu | sigma | Q | Age (centred) | Sex | DAS-28 |
| Mu | 0.00744 | -0.00102 | 0.005915 | 1.41E-05 | -0.00038 | -0.00091 |
| Sigma | -0.00102 | 0.001821 | -0.00304 | -2.2E-06 | -4.3E-05 | 7.78E-05 |
| Q | 0.005915 | -0.00304 | 0.021535 | 1.25E-05 | 0.000245 | -0.00045 |
| Age | 1.41E-05 | -2.2E-06 | 1.25E-05 | 1.3E-06 | -4E-06 | 0 |
| Sex | -0.00038 | -4.3E-05 | 0.000245 | -4E-06 | 0.001009 | 2.46E-05 |
| DAS-28 | -0.00091 | 7.78E-05 | -0.00045 | 0 | 2.46E-05 | 0.000123 |

| Table S33: NCT00361335: generalised gamma model coefficients | |
| --- | --- |
| Mu | -2.02391 |
| Sigma | -1.46475 |
| Q | 0.602766 |
| Age | 0.004842 |
| Sex | -0.07576 |
| DAS-28 | 0.085114 |

| Table S34: NCT00361335: variance covariance matrix from generalised gamma model | | | | | | |
| --- | --- | --- | --- | --- | --- | --- |
|  | Mu | sigma | Q | Age (centred) | Sex | DAS-28 |
| Mu | 0.006429 | -0.00076 | 0.00388 | -3.6E-05 | 1.24E-05 | -0.00061 |
| Sigma | -0.00076 | 0.001408 | -0.00239 | -3.3E-06 | -2.4E-05 | 8.55E-05 |
| Q | 0.00388 | -0.00239 | 0.015101 | 1.57E-05 | 0.000134 | -0.00043 |
| Age | -3.6E-05 | -3.3E-06 | 1.57E-05 | 8E-07 | -2.6E-06 | 0 |
| Sex | 1.24E-05 | -2.4E-05 | 0.000134 | -2.6E-06 | 0.00064 | 5E-07 |
| DAS-28 | -0.00061 | 8.55E-05 | -0.00043 | 0 | 5E-07 | 8.38E-05 |

| Table S35: NCT01316900: generalised gamma model coefficients | |
| --- | --- |
| Mu | -1.38005 |
| Sigma | -0.95181 |
| Q | 1.148042 |
| Age | -0.00065 |
| Sex | -0.13994 |
| FEV1 (% predicted) | -0.00288 |

| Table S36: NCT01316900: variance covariance matrix from generalised gamma model | | | | | | |
| --- | --- | --- | --- | --- | --- | --- |
|  | Mu | sigma | Q | Age (centred) | Sex | FEV1 (% predicted) |
| Mu | 0.003355 | -0.00033 | 0.001079 | -9E-07 | -0.00078 | -5.1E-05 |
| Sigma | -0.00033 | 0.00128 | -0.0022 | 1.6E-06 | -8.6E-05 | -3.9E-06 |
| Q | 0.001079 | -0.0022 | 0.009302 | -5.5E-06 | 0.000276 | 1.22E-05 |
| Age | -9E-07 | 1.6E-06 | -5.5E-06 | 2.2E-06 | -6.1E-06 | -1E-07 |
| Sex | -0.00078 | -8.6E-05 | 0.000276 | -6.1E-06 | 0.000878 | 5.2E-06 |
| FEV1 (% predicted) | -5.1E-05 | -3.9E-06 | 1.22E-05 | -1E-07 | 5.2E-06 | 0.000001 |

| Table S37: NCT01316913: generalised gamma model coefficients | |
| --- | --- |
| Mu | -1.35748 |
| Sigma | -1.05067 |
| Q | 1.255863 |
| Age | -0.00199 |
| Sex | -0.12404 |
| FEV1 (% predicted) | -0.00219 |

| Table S38: NCT01316913: variance covariance matrix from generalised gamma model | | | | | | |
| --- | --- | --- | --- | --- | --- | --- |
|  | Mu | sigma | Q | Age (centred) | Sex | FEV1 (% predicted) |
| Mu | 0.002384 | -0.00023 | 0.000707 | -6.3E-06 | -0.00051 | -3.7E-05 |
| Sigma | -0.00023 | 0.001323 | -0.00233 | -9E-07 | -5.4E-05 | -5.8E-06 |
| Q | 0.000707 | -0.00233 | 0.009506 | 2.6E-06 | 0.000171 | 1.81E-05 |
| Age | -6.3E-06 | -9E-07 | 2.6E-06 | 0.000002 | -3.7E-06 | 0 |
| Sex | -0.00051 | -5.4E-05 | 0.000171 | -3.7E-06 | 0.000663 | 2.4E-06 |
| FEV1 (% predicted) | -3.7E-05 | -5.8E-06 | 1.81E-05 | 0 | 2.4E-06 | 8E-07 |

| Table S39: NCT01957163: generalised gamma model coefficients | |
| --- | --- |
| Mu | -1.49634 |
| Sigma | -0.86352 |
| Q | 0.71645 |
| Age | -0.00257 |
| Sex | -0.11424 |
| FEV1 (% predicted) | -0.00284 |

| Table S40: NCT01957163: variance covariance matrix from generalised gamma model | | | | | | |
| --- | --- | --- | --- | --- | --- | --- |
|  | Mu | sigma | Q | Age (centred) | Sex | FEV1 (% predicted) |
| Mu | 0.004403 | -0.00033 | 0.001445 | -1.2E-05 | -0.00084 | -7E-05 |
| Sigma | -0.00033 | 0.001269 | -0.00201 | 0.000004 | -7.8E-06 | -5.5E-06 |
| Q | 0.001445 | -0.00201 | 0.011348 | -1.8E-05 | 3.84E-05 | 2.38E-05 |
| Age | -1.2E-05 | 0.000004 | -1.8E-05 | 4.6E-06 | -1.9E-06 | -3E-07 |
| Sex | -0.00084 | -7.8E-06 | 3.84E-05 | -1.9E-06 | 0.001276 | 4E-07 |
| FEV1 (% predicted) | -7E-05 | -5.5E-06 | 2.38E-05 | -3E-07 | 4E-07 | 1.7E-06 |

| Table S41: NCT02119286: generalised gamma model coefficients | |
| --- | --- |
| Mu | -1.49243 |
| Sigma | -0.88122 |
| Q | 1.07303 |
| Age | -0.00321 |
| Sex | -0.04148 |
| FEV1 (% predicted) | -0.00326 |

| Table S42: NCT02119286: variance covariance matrix from generalised gamma model | | | | | | |
| --- | --- | --- | --- | --- | --- | --- |
|  | Mu | sigma | Q | Age (centred) | Sex | FEV1 (% predicted) |
| Mu | 0.00484 | -0.00051 | 0.001709 | -2.2E-06 | -0.0009 | -7.6E-05 |
| Sigma | -0.00051 | 0.001849 | -0.00346 | 2.8E-06 | 2.91E-05 | -9.5E-06 |
| Q | 0.001709 | -0.00346 | 0.014391 | -9.2E-06 | -9.6E-05 | 3.13E-05 |
| Age | -2.2E-06 | 2.8E-06 | -9.2E-06 | 0.000004 | -8.6E-06 | -1E-07 |
| Sex | -0.0009 | 2.91E-05 | -9.6E-05 | -8.6E-06 | 0.001214 | 2.8E-06 |
| FEV1 (% predicted) | -7.6E-05 | -9.5E-06 | 3.13E-05 | -1E-07 | 2.8E-06 | 1.7E-06 |
